# Effects of Pirfenidone on Idiopathic Pulmonary Fibrosis Progression and Safety: Results of Multicenter Prospective Observational Study

**DOI:** 10.3390/life13020483

**Published:** 2023-02-10

**Authors:** Sergey Avdeev, Mikhail Ilkovich, Stanislav Terpigorev, Sergey Moiseev, Igor Tyurin

**Affiliations:** 1 Department of Pulmonology, Sechenov First Moscow State Medical University (Sechenov University), 119048 Moscow, Russia; 2 Institute for Interstitial and Orphan Lung Diseases, First Pavlov State Medical University of St. Petersburg, 197022 Saint Petersburg, Russia; 3 Moscow Regional Research and Clinical Institute (“MONIKI”), 129110 Moscow, Russia; 4 Tareev Clinic of Internal Diseases, Sechenov First Moscow State Medical University (Sechenov University), 119021 Moscow, Russia; 5 Russian Medical Academy for Postgraduate Education, 123242 Moscow, Russia

**Keywords:** pirfenidone, idiopathic pulmonary fibrosis, progression, safety

## Abstract

**Simple Summary:**

Idiopathic pulmonary fibrosis (IPF) commonly occurs in men in their 60s and results in replacement of the lungs parenchyma with fibrotic tissue. Usually, with such a diagnosis, patients die within 3–5 years. This disease is relatively rare, with approximately 1 case per 10,000 populations. Our study followed 55 patients who took pirfenidone, a medicine which can stop fatal changes in the lungs. This was a multicenter prospective study involving 11 expert centers in different parts of the country. Patients took the medicine and were examined in the usual way with regard to their real life conditions. For the efficacy assessment, we used not only the traditional outcomes (symptoms, pulmonary function, etc.), but also high-resolution computed tomography score and European Quality of Life 5-Dimension Questionnaire. In general, in 80–90% of patients, the disease did not progress. In the study, there was a very thorough evaluation of safety and tolerability of treatment with pirfenidone. Serious adverse effects were not registered when taking the medication.

**Abstract:**

The aim of this study was to determine the effectiveness of pirfenidone in patients with idiopathic pulmonary fibrosis (IPF) seen in clinical practice. Fifty-five adults with IPF were enrolled in this multicenter, open-label, non-randomized, non-controlled, interventional clinical study. All patients received pirfenidone 2403 mg/day (three 267 mg capsules three times daily) for 26 weeks. After 26 weeks of treatment, the mean change in absolute forced vital capacity (FVC) was 128.8 mL (95% confidence interval [CI] −26.8, 284.4) and the mean change in relative predicted FVC was −0.10% (95% CI −3.18, 2.99). Stable disease (defined as improvement of ≥0% or a decline of <10% to 0% of the corresponding FVC value) was observed in most patients (relative FVC, 90.9%; absolute FVC, 83.6%). There was no statistically significant change in the mean high-resolution computed tomography fibrosis score or lung opacity score at week 26 compared with baseline. Treatment-emergent adverse events were reported in 80% of patients during the treatment period; most of them were mild or moderate in severity. No serious pirfenidone-related adverse events were observed during the study period. Pirfenidone was generally safe and effective for controlling functional decline and stabilizing disease in patients with IPF encountered in clinical practice in Russia.

## 1. Introduction

Idiopathic pulmonary fibrosis (IPF) is a chronic, relentlessly progressive, irreversible, and fatal lung disease, with an average life expectancy of 3–5 years after diagnosis. IPF affects around 5 million people worldwide, disproportionately affects men, is more common with increasing age, and is increasing in prevalence [1,2]. The histopathological hallmarks of IPF are subpleural fibrosis, subepithelial myofibroblastic foci, and microscopic honeycombing [3,4].

Changes in clinical and physiological variables predict survival in patients with IPF. Forced vital capacity (FVC) is a reliable, valid, and reproducible measure of disease progression in these patients. Deterioration in FVC over time is a well-established predictor of mortality. In patients with IPF, a ≥10% decline in FVC predicted (%pred) over a 12-month period is associated with significantly lower 5-year survival [5,6]. Another strong predictor of mortality in patients with IPF is an objective decline in functional exercise capacity (e.g., the 6-min walk distance [6MWD]). The minimal clinically important difference in 6MWD in patients with IPF has been defined as 22–45 m [7,8,9,10,11].

Pirfenidone is a pyridone derivative with anti-inflammatory, anti-fibrotic, and antioxidant properties and is approved worldwide for the treatment of IPF based on its ability to slow functional decline and disease progression, as shown in several pivotal clinical trials [12,13,14,15]. Although its precise mechanism of action is unknown, pirfenidone has been found to achieve a significant reduction in the proportion of patients with a > 10% decline in FVC during 52 weeks of follow-up, to increase the 6MWD, and to improve progression-free survival when compared with placebo. Pooled analysis of the combined patient populations from the three global randomized Phase III trials of pirfenidone (*n* = 623) versus placebo (*n* = 624) showed that the relative risk of death for all four mortality outcomes at week 52 was significantly lower in the pirfenidone group than in the placebo group (all-cause mortality, hazard ratio [HR] 0.52, 95% confidence interval [CI] 0.31–0.87; treatment-emergent all-cause mortality, HR 0.45 95% CI 0.24–0.83; IPF-related mortality, HR 0.35, 95% CI 0.17–0.72; and treatment-emergent IPF-related mortality, HR 0.32, 95% CI 0.14–0.76) [16].

In Russia, owing to the lack of epidemiological studies, only rough estimates of the number of patients diagnosed with IPF are available. It has been suggested that the prevalence and incidence of IPF are approximately 9–11 and 4–6 cases per 100 000 populations, respectively [17]. Russian Respiratory Society guidelines recommend pirfenidone as a specific evidence-based treatment for IPF [18]. Nonetheless, at the stage of clinical development of pirfenidone, no clinical centers in Russia were included in the global clinical trials programme, and experience with pirfenidone in clinical practice has been limited. Therefore, a local trial was needed to estimate the effectiveness of pirfenidone in the Russian population with IPF.

The purpose of this study was to estimate the effectiveness of pirfenidone 267 mg capsules (Esbriet^®^; Catalent Pharma Solutions, Somerset, NJ, USA) in patients with IPF seen in clinical practice in Russia.

Trial registration: ClinicalTrials.gov identifier NCT03208933.

## 2. Materials and Methods

### 2.1. Study Design and Ethical Considerations

**Figure 1:**
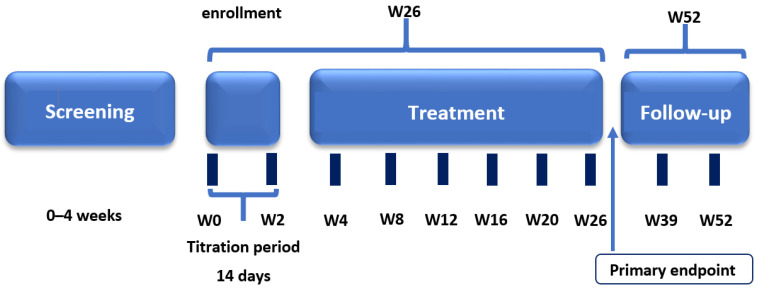
Clinical study design.

This prospective, open-label, multicenter, non-randomized, non-controlled clinical study was conducted at 11 investigational centers in the Russian Federation. The study consisted of four periods: screening and washout (up to 4 weeks); treatment (26 weeks including a 2-week titration period); post-treatment follow-up (2–4 weeks), and long-term follow-up for further assessment in patients who continued treatment with pirfenidone in clinical practice (Figure 1). Clinical study visits were conducted on day –28 to day –7 (screening), day 1 (start of treatment), and weeks 1, 2, 4, 8, 12, 16, 20, 26, 28–30, 39, and 52.

The primary efficacy endpoints were the changes from baseline to week 26 in absolute FVC (mL) and relative FVC (%pred) measured using spirometry. During screening, spirometry measurements were obtained before and after the administration of salbutamol (albuterol) from a metered-dose inhaler. The bronchodilator test was not performed during subsequent visits.

The global and integrated responses of all body systems involved during walking were assessed using the 6MWD based on the 2002 American Thoracic Society recommendations [19]. At the start and end of the 6-min exercise test, the patient graded his/her breathing level by answering the question “Please rate the current severity of your breathlessness by circling the most appropriate number on the following scale”. Severity was rated on the Borg scale [20].

Semi-quantitative analysis of the HRCT scans was performed by a local radiologist and reviewed by a central reader using a quantitative scale, as previously described by Oda et al. [21]. HRCT scans were acquired using the same protocol during the study period. HRCT fibrosis and ground-glass (GG) opacity scores were evaluated. The radiologist assessed the four main HRCT findings: (1) areas with normal attenuation, (2) reticular abnormality, (3) traction bronchiolectasis or bronchiectasis, and (4) honeycombing. The extent of involvement of each abnormality was assessed independently for each of three zones of each lung. The scores for the six zones were averaged to determine the total fibrosis score for each patient. The scores for the six zones were averaged to determine the total fibrosis score for each patient. The sign “GG opacity” was qualified separately, and its change over time was assessed outside the framework of a total summed index of fibrosis (HRCT fibrosis score). GG opacity was graded as reticular abnormality to calculate the score for each zone, that is, the percentage area of opacity in a zone was multiplied by a score of 2.

The European Quality of Life (EuroQol) 5-Dimension Questionnaire, 5-level version (EQ-5D-5L), was used as a self-report health status questionnaire [22]. The EuroQol EQ-5D has two components: (1) a five-item health state profile that assesses mobility, self-care, usual activities, pain/discomfort, and anxiety/depression, and (2) a visual analogue scale (VAS) that measures health state. Published weighting systems allow creation of a single summary score. Overall scores range from 0 to 1, with low scores representing a higher level of dysfunction.

Safety and tolerability were evaluated throughout the study. All AEs observed by the investigator, elicited during study visits or spontaneously reported by the patient, were collected and classified according to the Medical Dictionary for Regulatory Activities version 22.1. Laboratory results, vital signs, and findings on physical examination and a 12-lead ECG were obtained for all patients at baseline and post-baseline visits to the study site. Routine laboratory investigations (serum hematology and biochemistry, urinalysis, and a pregnancy test if applicable) were performed in local laboratories at each study site.

Patient eligibility was confirmed during screening. Eligible patients were assigned to receive pirfenidone 2403 mg/day administered orally in divided doses three times daily (TID) with food. The dose was titrated over 14 days (days 1–7, one capsule TID; days 8–14, two capsules TID) to the full dose of nine capsules per day (three 267 mg capsules TID). Patients remained on a stable maintenance dose for the duration of the study period unless the dose was reduced to manage adverse events (AEs) or titrated again when the study treatment was restarted after an appropriate interruption.

All prohibited therapies, including therapy targeted to treat IPF, were discontinued at least 28 days before the start of the treatment period. Patients were not allowed to receive any treatment for IPF other than long-term corticosteroid therapy as part of routine management of IPF (maximum 15 mg/day of prednisolone or equivalent). Corticosteroids were also permitted for use without dose restriction for up to 21 days in patients experiencing an acute exacerbation of IPF. The study drug was continued during exacerbations if possible.

The study was approved by the Russian Federation Ministry of Health and the independent ethics committee at each study site. The study was conducted in accordance with the International Council for Harmonization Good Clinical Practice guidelines, local regulatory requirements, and the principles embodied in the Declaration of Helsinki.

### 2.2. Subjects and Inclusion/Exclusion Criteria

Adult male or female patients aged 40–80 years with IPF, defined by a clinical, radiological, or pathological pattern of usual interstitial pneumonia (UIP), were eligible to participate in the study. Key inclusion criteria were compliance with the American Thoracic Society/European Respiratory Society diagnostic criteria for IPF [12], presence of symptoms for at least 6 months before screening, abnormal lung function (%FVC ≥40%pred and carbon monoxide diffusing capacity (%DLCO) ≥30%pred), a 6MWD ≥100 m, no features supporting an alternative diagnosis on transbronchial biopsy, bronchoalveolar lavage, or surgical lung biopsy, if performed.

Patients were ineligible if they had any of the following: predicted life expectancy <12 months (in the investigator’s opinion) or planned lung transplantation during the study period; cigarette smoking within 28 days before the start of treatment or unwillingness to avoid tobacco products during the study; history of clinically significant environmental exposure known to cause pulmonary fibrosis; known explanation for interstitial lung disease (such as radiation, drug toxicity, sarcoidosis, hypersensitivity pneumonitis, organizing pneumonia, human immunodeficiency virus infection, or cancer); any connective tissue disease (including scleroderma, polymyositis/dermatomyositis, systemic lupus erythematosus, rheumatoid arthritis); significant concomitant emphysema (according to baseline high-resolution computed tomography (HRCT) scans); history of severe hepatic or renal impairment; unstable or deteriorating cardiac or pulmonary (other than IPF) disease within the previous 6 months; severe laboratory abnormalities at baseline; and an electrocardiogram (ECG) with a QT interval corrected according to Frideric formula of >500 ms.

### 2.3. Statistical Analysis

Sample size estimation was based on the feasibility of subject enrolment using a precision-based approach [23]. In the pivotal ASCEND study, the primary efficacy endpoint (FVC, %pred) in the pirfenidone group decreased from a mean ± standard deviation of 67.8 ± 11.24 at baseline to 65.3 ± 14.52 at week 26, with stable disease (decline in FVC <10% to 0%) in 60.1% of study subjects [15]. The sample size required for a two-sided 95% CI with a margin of error of 0.125, assuming a proportion of patients with stable disease of 0.601, was calculated to be 60. With a sample size of 60 patients, an expected mean value of 2.5%, and a standard deviation of 20, the distance from the mean to the limit of the two-sided 95% CI for the mean change from baseline to week 26 (FVC, %pred) extends 5 (margin of error).

The treated set (TS) consisted of all patients who were allocated to treatment and received any dose of the study medication. The full analysis set (FAS) consisted of all patients in the TS who had data available for at least one post-baseline assessment of any efficacy measurement. The complete case set (CCS) included patients in the FAS for whom data for the primary efficacy endpoint (FVC at week 26) were available. FAS was the main set used for the efficacy analysis and the CCS was used for the sensitivity analysis. The safety data were analyzed in the TS.

Data on FVC, performance on the 6MWD, EQ-5D-5L index score, VAS score, individual dimensions (mobility, self-care, usual activities, pain/discomfort, anxiety/depression), and HRCT fibrosis and lung opacity scores at baseline were analyzed using standard descriptive statistics for continuous variables and the 95% CI for mean values and mean changes. The distribution (number and percentage) of performance on the 6MWD across the three categories of change from baseline (decline of ≥50 m or death before week 26; decline of <50 m to 0 m (exclusively); improvement of ≥0 m) was provided. Changes in the HRCT fibrosis score from baseline to week 26 were examined using the paired t-test. The type I error rate was set to 5%.

The baseline FVC and 6MWD values were the averages of the measurements obtained at the screening and day 1 visits; if either was missing, the non-missing value was used. The FVC and 6MWD values at week 26 were the average of the measurements obtained on two separate days. If either was missing, the non-missing value was used.

In the primary analysis of the primary outcome variable (change in FVC in both mL and %pred) from baseline to week 26 in the FAS, missing data due to death were replaced with the worst possible value (FVC = 0 mL or 0%). Data that were missing for reasons other than death were replaced with imputed values based on the average measurements for “similar” patients at that time point. After imputation, the values were further categorized according to change from baseline (decline of ≥10% or death before week 26; decline of <10% to 0%; improvement of ≥0%), and the number and percentage of patients in each category were tabulated. Other values were not imputed.

The study data are presented as the mean and standard deviation, the percentage, or number and percentage as appropriate. All statistical analyses were performed using SAS software (version 9.4; SAS Institute Inc., Cary, NC, USA).

### 2.4. Patient and Public Involvement

Neither patients nor the public were involved in the study design or conduct, and thus will not be involved with the reporting or dissemination plans of the research.

## 3. Results

### 3.1. Baseline Patient Characteristics

**Figure 2:**
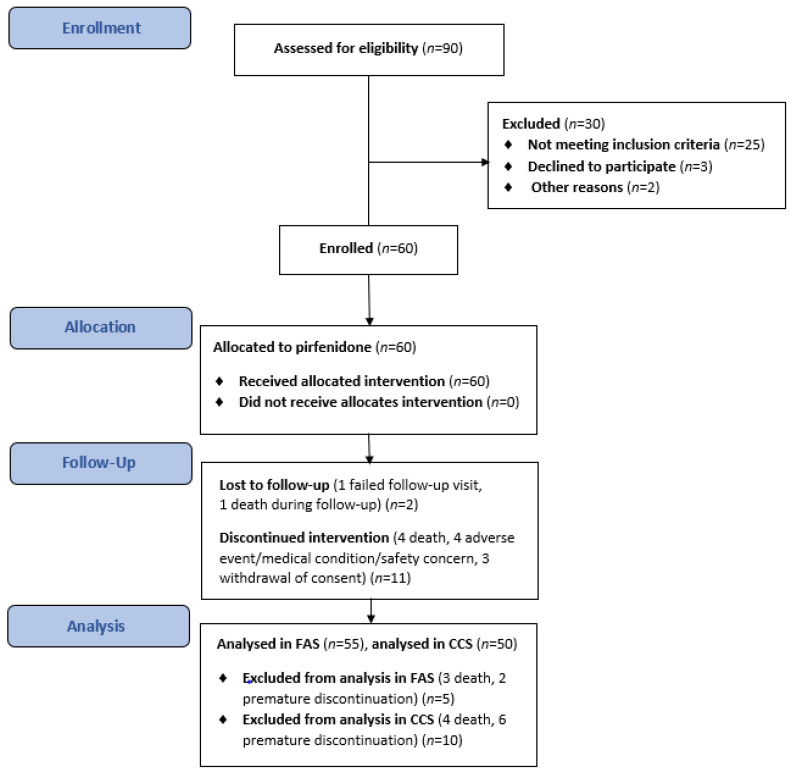
The flowchart of the study.

A total of 60 patients with IPF received treatment with pirfenidone over the course of this evaluation between October 2017 and November 2019. Details of the patient assessment and enrolment process, dropouts, and outcomes are presented in Figure 2. Sixty patients were enrolled and participated in the main component of the study; 47 patients (78.3%) completed the study, 13 (21.7%) terminated prematurely, and 8 (13.3%) continued treatment in the rollover phase. All patients allocated to treatment were included in the TS, 55 (91.7%) were included in the FAS, and 50 (83.3%) were included in the CCS. Five patients were excluded from the FAS (death, *n* = 3; discontinuation, *n* = 2) before any post-baseline efficacy assessment. A further five patients were not included in the CCS because of discontinuation (*n* = 4) or death (*n* = 1) before the 26-week assessment. Table 1 shows the baseline demographic and clinical characteristics of IPF patients in the TS.

The mean patient age was 67.4 ± 7.7 years and 68% of patients were male. The mean duration of clinical symptoms consistent with IPF was 24.9 ± 17.4 months (median, 21.5 months). Most concurrent medical conditions were vascular (71.7%), cardiac (28.3%), or gastrointestinal (28.3%). The most common concomitant medications were antithrombotic agents (36.7%), beta- blockers (36.7%), agents for peptic ulcer and gastroesophageal reflux disease (33.3%), systemic corticosteroids (26.7%), oxygen (20%), expectorants (20%), and angiotensin-converting enzyme inhibitors (18.3%).

### 3.2. Treatment Efficacy

**Figure 3:**
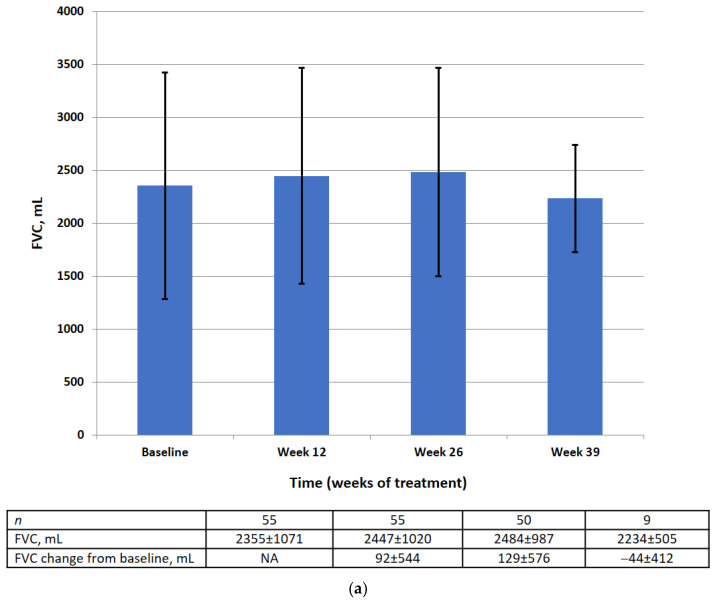

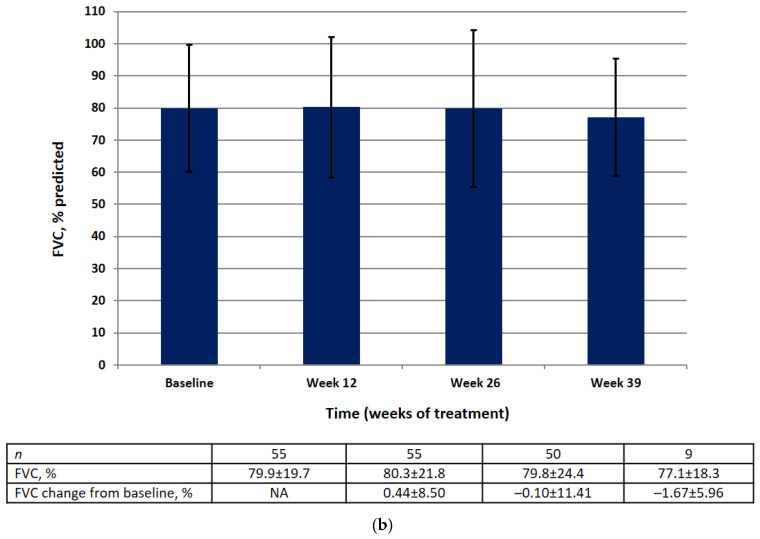
FVC data (FAS) at baseline, week 12, week 26, week 39 in the absolute (mL) (**a**) and relative (% pred.) (**b**). Data are presented as mean ± SD.

Absolute and relative FVC values by visit are shown in Figure 3. There was no significant difference in the mean absolute or relative FVC value at weeks 12, 26, or 39. The results of the statistical analysis for the primary efficacy endpoint are summarized in Table 2.

At baseline in the FAS, the mean absolute FVC was 2355 mL (95% CI 2065–2644) and the mean relative FVC was 79.89%pred (95% CI 74.56–85.22). There was no significant change in the mean absolute FVC (128.8 mL, 95% CI −26.8, 284.4) or the mean relative FVC (−0.10%, 95% CI −3.18, 2.99) between baseline and week 26.

Most of the patients (83.63%) showed an improvement of ≥0% or a decline of <10% to 0% in absolute FVC from baseline and 16.4% showed a decline of ≥10%. A majority (90.9%) had an improvement of ≥0% or a decline of <10% to 0% in relative FVC from baseline and 9.1% showed a decline of ≥10%.

**Figure 4:**
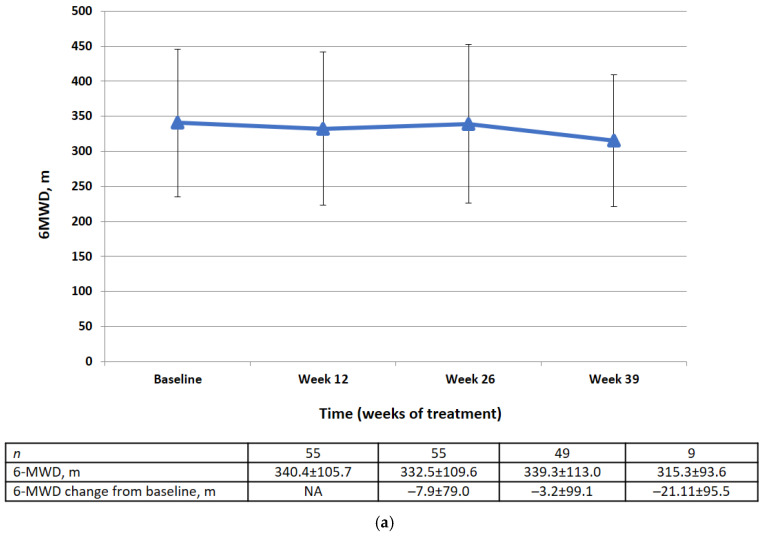

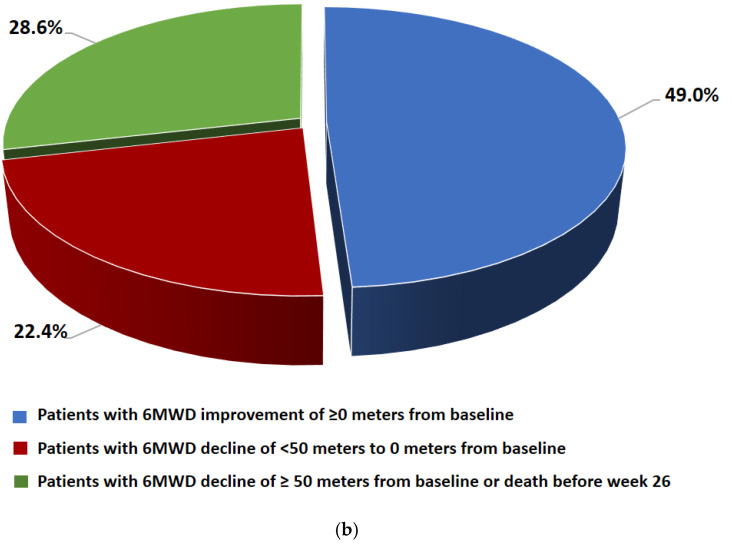
(**a**) 6MWD data (FAS) at baseline, week 12, week 26, week 39 (m). Data are presented as mean ± SD. (**b**) Changes in 6MWD from the baseline to week 26 by category (FAS).

Changes in 6MWD from baseline by visit and category at week 26 are shown in Figure 4. There was no significant difference in the mean change in 6MWD between baseline and week 26 (−3.2 ± 99.1 m). At week 26, 49% of the patients had an improvement in 6MWD of ≥0 m from baseline, 22.4% had a decline of <50 m to 0 m from baseline, and 28.6% had a decline of ≤50 m from baseline or died before week 26.

There was no statistically significant change in HRCT lung opacity and fibrosis scores at week 26 compared with baseline (Table 3). The mean HRCT fibrosis score was 159.5 ± 40.6% at baseline and 159.2 ± 40.9% at week 26. The difference (1.9 ± 8.4) was not statistically significant (*p* = 0.1331). The mean HRCT lung opacity score was 28.3 ± 18.0 at baseline and 29.6 ± 20.7 at week 26. The mean change of 1.7 ± 8.0 was not statistically significant.

The responses to the EQ-5D-5L questionnaire after 26 weeks of treatment are summarized in Table 4. Compared with baseline, there was no significant change in the mean VAS score (−0.6 ± 17.2) or in the mean index score (−0.03 ± 0.18) by week 26. Furthermore, there were no significant changes in individual dimensions (mobility, self-care, usual activities, pain/discomfort, and anxiety/depression).

### 3.3. Treatment Safety

The median overall treatment duration was 49.9 weeks (study period, 26 weeks; rollover phase, 24 weeks). The mean number of pirfenidone capsules taken during the study period was 1437 ± 434 (range 198–2625). Forty-eight patients (80%) reported at least one treatment-emergent AE during the treatment period (Table 5).

AEs reported during the treatment period were of grade 1 or 2 severity in 61.7% of cases, maximal grade 5 (fatal) in 8.3%, maximal grade 4 (life-threatening or requiring urgent intervention) in 3.3%, and maximal grade 3 (severe or medically significant) in 6.7%. Thirteen treatment-emergent serious adverse events (SAEs) in nine patients (15%) were registered during the treatment period. There were no reports of SAEs related to the study medication.

During the study period, 10 deaths were registered for patients who signed informed consent. Three of these deaths occurred during the screening period, five during the treatment period (unexplained, *n* = 2; progression of IPF, *n* = 2; hospital pneumonia, *n* = 1), one during the follow-up period after the treatment period (IPF exacerbation), and one during the rollover phase (sudden cardiac death).

The most common treatment-emergent AEs during the treatment period were nausea (in 26.7% of patients), decreased appetite (21.7%), dyspepsia (13.3%), vomiting (13.3%), diarrhea (11.7%), and dyspnea (10%). Vital signs, findings on physical examination, laboratory tests, and ECG assessments were normal or non-significantly abnormal clinically.

## 4. Discussion

This prospective multicenter clinical study was designed to estimate the effectiveness of pirfenidone in patients with IPF seen in clinical practice in Russia. The primary efficacy analysis found no significant deterioration in relative or absolute FVC between baseline and week 26 in treated patients. Stable disease (defined as improvement of ≥0% or a decline of <10% to 0% of the corresponding FVC value) was observed in most of the patients. There was no significant change in relative FVC in 90.9% of patients and no significant change in absolute FVC in 83.6%. At the same time, in patients not receiving antifibrotic therapy, the decrease in FVC within 6 months should be at least 110 mL [24,25]. There was no significant difference in the 6MWD or EQ-5D-5L questionnaire scores between baseline and week 26, which is consistent with the primary efficacy results. Moreover, there were no statistically significant changes in the mean HRCT fibrosis and lung opacity scores between baseline and week 26. Exacerbations occurred in 7.3% of patients during the treatment period.

Pirfenidone was safe and generally well tolerated. No SAEs related to pirfenidone were observed in this study. The type and frequency of AEs were consistent with the known safety profile of pirfenidone, including gastrointestinal events, which were the most frequent treatment-emergent AEs. AEs reported during the treatment period were typically mild or moderate in severity, and few led to discontinuation of treatment. There were five deaths during the treatment period, two of which were due to progression of IPF and three for other reasons.

The efficacy of pirfenidone in patients with IPF demonstrated in the present study is in line with the findings of other Phase III trials reported individually and in meta-analysis [12,13,14,15,16]. In our study, disease progression (defined as a decline in relative FVC of ≥10%) was evident in 9.1% of pirfenidone-treated patients in the FAS at week 26. A similar rate of disease progression (6.7%) was reported in the pooled analysis of the ASCEND and CAPACITY trials at month 12 in the intention-to-treat population. Several real-life observational cohort studies also confirmed that the observed decline in FVC and the safety profile during pirfenodone therapy were consistent with the findings of the randomized trials [26,27,28]. Patients with IPF could be categorized as rapid or slow progressors according to whether their decline in FVC during the year preceding treatment with pirfenidone was >10% or ≤10% predicted. The magnitude of the effect of pirfenidone on FVC may vary greatly between these subgroups [6]. Patients with rapidly and relatively stable/slowly progressive IPF are thought to have distinct gene expression and inflammatory profiles in the lung parenchyma [29,30,31]. However, given that there is no universally agreed definition of these two clinical IPF phenotypes, we present the results for our entire study population.

Our rate of discontinuation of pirfenidone because of AEs was lower than that in CAPACITY and ASCEND (8.3% vs. 14.8% and 14.4%, respectively), which confirms the safety and tolerability of the drug in clinical practice [14,15]. The preferred approach for managing the most frequent treatment-emergent gastrointestinal AEs is temporary dose reduction or interruption followed by a slow titration back to the full or previously prescribed dose [32]. Taking pirfenidone with a substantial amount of food, spacing ingestion of the three tablets between courses, and the use of prokinetic agents or proton pump inhibitors may also reduce the rate of absorption of pirfenidone and help control adverse gastrointestinal effects [33].

Despite our clear results regarding the efficacy of pirfenidone, there are some limitations that should be borne in mind when interpreting or generalizing the findings of this study. First, there was no way to establish the assay sensitivity of the study because a placebo effect could not be delineated. Second, the open-label trial design means that the possibility of response bias cannot be excluded. However, the main efficacy parameters are objective, and the selected primary endpoint of irreversible loss of lung function is clinically relevant and used widely in clinical practice. Therefore, the results of the primary efficacy analysis provide strong evidence that pirfenidone reduces the decline in lung function in patients with IPF despite the limitations arising from the non-comparative, open-label design of the study. Third, the number of patients in the study was relatively small, although the studied patients were managed by different expert centers and quite well reflect the population of IPF patients in our country. Finally, the treatment period in our study was relatively short. The duration of our study was 52 weeks, but the primary endpoint, change in FVC, was assessed at week 26. Most of the landmark studies lasted at least one year. However, in several well-known, randomized [34,35], and real-life observational studies [36,37], the study duration was 24–26 weeks. This time turned out to be quite enough to demonstrate significant changes in FVC. For example, in a study by Mahler et al., the difference in FVC measured by in-hospital spirometry between patients treated with pirfenidone and placebo was statistically significant at 24 weeks [35].

## 5. Conclusions

The findings of this clinical study confirm that administration of pirfenidone 2403 mg/day is an effective treatment option for controlling functional decline and stabilizing disease in patients with IPF in clinical practice in Russia. Pirfenidone was generally well tolerated and had an acceptable safety profile without any unexpected safety findings or any clinically meaningful differences from the published data.

## Figures and Tables

**Table 1:** Demographic and baseline main clinical characteristics of IPF patients (TS).

Parameter	Total (*n* = 60)
Age (years), mean ± SD	67.4 ± 7.7
Sex (male), n (%)	41 (68)
Body mass index (kg/m2), mean ± SD	28.6 ± 4.2
Duration of clinical symptoms consistent with IPF (months)	
mean ± SD	24.9 ± 17.4
median	21.5
minimum—maximum	6–84
HRCT scan results at screening	
regional CT specialist: definitive UIP pattern, *n* (%)	33 (55)
regional CT specialist: possible UIP pattern, *n* (%)	27 (45)
central CT specialist: definitive UIP pattern, *n* (%)	20 (33)
central CT specialist: possible UIP pattern, *n* (%)	39 (65)
Spirometry parameters at screening	
pre-bronchodilator FEV1/FVC, mean ± SD	0.839 ± 0.064
post-bronchodilator FEV1/FVC, mean ± SD	0.845 ± 0.081
pre-bronchodilator FVC (absolute) in mL, mean ± SD	2333.1 ± 1118.9
post-bronchodilator FVC (absolute) in mL, mean ± SD	2354.9 ± 1153.0
pre-bronchodilator FVC (relative) in % pred., mean ± SD	80.3 ± 19.9
post-bronchodilator FVC (relative) in % pred., mean ± SD	80.8 ± 21.0
GAP index	
mean± SD	3.6 ± 1.4
stage I, *n* (%)	30 (50)
stage II, *n* (%)	23 (38)
stage III, *n* (%)	7 (12)
Diffusing capacity (DLCO)	
predicted %, mean ± SD	45.5 ± 12.9

SD = standard deviation; CT = Computed tomography; UIP = Usual interstitial pneumonia; FVC= Forced vital capacity; FEV1 = Forced expiratory volume in the first second.

**Table 2:** Analysis of primary efficacy endpoint—change from baseline to week 26 in the absolute (ml) and relative (% pred.) FVC (FAS).

Analysis	FAS (*n* = 55)	CCS (*n* = 50)
FVC (absolute), mL		
Baseline		
Mean [95% CI]	2355 [2065; 2644]	2366 [2053; 2679]
Week 26		
Mean [95% CI]	2484 [2217; 2750]	2546 [2277; 2814]
Mean change from the baseline [95% CI]	128.8 [−26.8; 284.4]	180.0 [25.5; 334.4]
Categorical (number of patients with), n (%)		
decline of ≥10% from baseline or death before week 26	9 (16.4)	7 (14.0)
decline of < 10% to 0%	17 (30.9)	16 (32.0)
improvement of ≥0% from baseline	29 (52.7)	27 (54.0)
FVC (relative), % pred.		
Baseline		
Mean [95% CI]	79.89 [74.56; 85.22]	80.41 [74.82; 86.00]
Week 26		
Mean [95% CI]	79.79 [73.21; 86.38]	81.49 [75.08; 87.90]
Mean change from the baseline [95% CI]	−0.10 [−3.18; 2.99]	1.08 [−1.56; 3.72]
Categorical (number of patients with), *n* (%)		
decline of ≥10% from baseline or death before week 26	5 (9.1)	3 (6.0)
decline of < 10% to 0%	25 (45.5)	23 (46.0)
improvement of ≥0% from baseline	25 (45.5)	24 (48.0)

FVC = Forced vital capacity; CI = confidence interval.

**Table 3:** High-resolution computed tomography (FAS): changes from baseline to week 26.

Assessment	Baseline (*n* = 52)	Week 26 (*n* = 47)
HRCT fibrosis score		
mean ± SD	159.5 ± 40.6	159.2 ± 40.9
change from baseline, mean ± SD	NA	1.9 ± 8.4
*p*-value		0.133
HRCT GG opacity score		
Mean ± SD	28.3 ± 18.0	29.6 ± 20.7
change from baseline, mean ± SD	NA	1.7 ± 8.0
*p*-value		0.235

HRCT= High-resolution computed tomography; GG = ground glass; SD = Standard deviation; Baseline is the last non-missing assessment prior to the first dose of the study drug.

**Table 4:** EQ-5D-5L (FAS)—change from baseline to week 26.

Assessment	Baseline (*n* = 55)	Week 26 (*n* = 48)
Parameter: VAS score		
mean ± SD	55.5 ± 11.6	55.0 ± 18.7
change from baseline, mean ± SD	NA	−0.6 ± 17.2
*Parameter: Index score*		
mean ± SD	0.57 ± 0.18	0.55 ± 0.24
change from baseline, mean ± SD	NA	−0.03 ± 0.18
Dimension: Mobility		
Level 1 (no problem), *n* (%)	6 (10.9)	3 (6.3)
Level 2 (slight problems), *n* (%)	11 (20.0)	10 (20.8)
Level 3 (moderate problems), *n* (%)	28 (50.9)	21 (43.8)
Level 4 (severe problems), *n* (%)	10 (18.2)	13 (27.1)
Level 5 (unable to walk about), *n* (%)	0	1 (2.1)
Dimension: Self-Care		
Level 1 (no problem), *n* (%)	16 (29.1)	12 (25.0)
Level 2 (slight problems), *n* (%)	15 (27.3)	13 (27.1)
Level 3 (moderate problems), *n* (%)	21 (38.2)	17 (35.4)
Level 4 (severe problems), *n* (%)	3 (5.5)	6 (12.5)
Level 5 (unable to wash or dress myself), *n* (%)	0	0
Dimension: Usual Activities		
Level 1 (no problem), *n* (%)	5 (9.1)	2 (4.2)
Level 2 (slight problems), *n* (%)	14 (25.5)	10 (20.8)
Level 3 (moderate problems), *n* (%)	30 (54.5)	28 (58.3)
Level 4 (severe problems), *n* (%)	5 (9.1)	4 (8.3)
Level 5 (unable to do my usual activities), *n* (%)	1 (1.8)	4 (8.3)
Dimension: Pain/Discomfort		
Level 1 (no pain or discomfort), n (%)	12 (21.8)	12 (30.0)
Level 2 (slight pain or discomfort), *n* (%)	15 (27.3)	11 (27.5)
Level 3 (moderate pain or discomfort), *n* (%)	23 (41.8)	15 (37.5)
Level 4 (severe pain or discomfort), *n* (%)	5 (9.1)	1 (2.5)
Level 5 (extreme pain or discomfort), *n* (%)	0	1 (2.5)
Dimension: Anxiety/Depression		
Level 1 (not anxious or depressed), *n* (%)	15 (27.3)	13 (27.1)
Level 2 (slight anxious or depressed), *n* (%)	20 (36.4)	17 (35.4)
Level 3 (moderate anxious or depressed), *n* (%)	16 (29.1)	14 (29.2)
Level 4 (severe anxious or depressed), *n* (%)	4 (7.3)	3 (6.3)
Level 5 (extremely anxious or depressed), *n* (%)	0	1 (2.1)

Note: VAS = Visual analogue scale; SD = Standard deviation; *n* (%)—the number and percentage of patients within a specific category. Baseline is the last non-missing assessment prior to the first dose of the study drug.

**Table 5:** Treatment-Emergent AEs During Treatment Period, Including Individual AEs Occurring in ≥5% of Subjects (TS).

Event, *n* (%)	Total (*n* = 60)
Serious treatment-emergent AE	9 (15.0%)
Treatment-emergent AEs leading to death	5 (8.3%)
Treatment-emergent AEs leading to discontinuation of study treatment	5 (8.3%)
Any treatment-emergent AE	48 (80.0%)
Severity Grade 1	12 (20.0%)
Severity Grade 2	25 (41.7%)
Severity Grade 3	4 (6.7%)
Severity Grade 4	2 (3.3%)
Severity Grade 5	5 (8.3%)
Treatment-emergent AEs related to study treatment	32 (53.3%)
Severity Grade 1	11 (18.3%)
Severity Grade 2	18 (30.0%)
Severity Grade 3	3 (5.0%)
Severity Grade 4	0
Severity Grade 5	0
Most frequent individual treatment-emergent AEs ( ≥ 5% of subjects)	
Nausea	16 (26.7%)
Decreased appetite	13 (21.7%)
Dyspepsia	8 (13.3%)
Vomiting	8 (13.3%)
Diarrhoea	7 (11.7%)
Dyspnea	6 (10.0%)
Cough	4 (6.7%)
Pruritus	4 (6.7%)
Weight decreased	4 (6.7%)
Abdominal discomfort	3 (5.0%)
Constipation	3 (5.0%)
Bronchitis	3 (5.0%)
Respiratory tract infection viral	3 (5.0%)
Rash	3 (5.0%)
Dizziness	3 (5.0%)
Dysgeusia	3 (5.0%)
Headache	3 (5.0%)

The table displays numbers (*n*) and percentages (based on *n*) of subjects with adverse events. Adverse events coded using MedDRA version 22.1.

## Data Availability

Not applicable.
